# Cheap Science

**Published:** 1867-08

**Authors:** 


### CHEAP SCIENCE.

We recently noticed the advertisements of two medical colleges. Each school has eight or ten professors and teachers. They each propose a course of five months, giving as many lectures each day as the students can listen to with profit. For this they propose to charge FORTY DOLLARS.

Our first thought was, that this is *fraudulently* cheap. But we must not judge. These are all experienced teachers. Some of them have been teaching these many years. They ought, by this time, to know what their services are worth. They once charged higher than this; and if they found, by experience and observation, that the fees were too high for the services, it was their duty to lower them. But, as "time is money," can students afford to spend five precious months in listening to instructions worth but eight dollars a month, though imparted by half a score of men? Either these men have undervalued their teachings, or students who attend on them undervalue their time.

But these teachers may suggest that we attend to our own business; which is just what we are doing. Educators cannot belittle their calling, without doing injustice to other educators.

"But dwellers in glass houses must not throw stones." We have recently heard of a Dental college proposing to give a student a course, and let him pay for it after he earns the money by practice. Whether or not he is to "board around" with the professors, during the session, on the same terms, we have not learned, but presume he is, as they are generous fellows, and do not profess to do things by halves. W.

